# Chromosome-level genome assembly of the Siberian chipmunk (*Tamias sibiricus*)

**DOI:** 10.1038/s41597-022-01910-5

**Published:** 2022-12-24

**Authors:** Ran Li, Mingfei Zhang, Muha Cha, Jishan Xiang, Xianfeng Yi

**Affiliations:** 1 School of Life Sciences, Qufu Normal University, Qufu, 273165 China; 2 Key Laboratory of Agro-Ecological Protection & Exploitation and Utilization of Animal and Plant Resources in Eastern Inner Mongolia, Chifeng University, Chifeng, 024000 China

**Keywords:** Genome, Zoology

## Abstract

*Tamias sibiricus* is regarded as one predominant scatter-hoarder that stores their food items both in small scattered caches and underground larder-hoards. This unique behavior, though providing essential seed dispersal services for many plant species worldwide, relies highly on accurate spatial memory and acute sense of olfaction. Here, we assembled a chromosome-scale genome of *T. sibiricus* using Illumina sequencing, PacBio sequencing and chromosome structure capture technique. The genome was 2.64 Gb in size with scaffold N50 length of 172.61 Mb. A total of 2.59 Gb genome data was anchored and orientated onto 19 chromosomes (ranging from 28.70 to 222.90 Mb) with a mounting rate of up to 98.03%. Meanwhile, 25,311 protein-coding genes were predicted with an average gene length of 32,936 bp, and 94.73% of these genes were functionally annotated. This reference genome will be a valuable resource for in-depth studies on basic biological possess and environmental adaptation of the Siberian chipmunk, as well as promoting comparative genomic analyses with other species within Rodentia.

## Background & Summary

The Siberian chipmunk, *Tamias sibiricus* (Laxmann, 1769) belongs to the subfamily Xerinae, within the family Sciuridae of the order Rodentia^1^. This species is a small, diurnal and ground-dwelling squirrel that lives in mountain and forest habitats with bushy understory^2^. The wild populations of *T. sibiricus* are naturally distributed in Russia and several east Asian countries (China, Mongolia, Korea and Japan). Meanwhile, this squirrel is one of most popular companion animals because of its attractive appearance and unique behavior^3^. Hence, it has been introduced as pets into European countries for decades and the accidentally escaped individuals have successfully established their populations in the wild^4^. Additionally, as important seed dispersal agents adopting the primary strategies of scatter- and larder-hoarding behavior, *T. sibiricus* provides essential seed dispersal services in many ecosystems across the world^5^. Over the past decades, studies of *T. sibiricus* have mainly focused on biology, behavior, ecology, and phylogeography^5–8^. However, little is known about the genetic basis and mechanism of its environmental adaptation because of limited molecular information.

In the present study, we constructed a high-quality genome assembly for the Siberian chipmunk using the integration of short reads (Illumina sequencing), long reads (PacBio sequencing) and Hi-C reads (proximity ligation chromatin conformation capture). The final assembled genome size of *T. sibiricus* was 2.64 Gb with the scaffold N50 length of 172.61 Mb. A total of 2.59 Gb assembled genome sequences were successfully anchored on 19 chromosomes. This number of chromosomes was consistent with the outputs of the karyotype ananlysis^9^. 1.03 Gb repetitive sequences were identified, constituting 38.87% of this reference genome. A total of 25,311 protein-coding genes were predicted, and 97.69% of these genes were functionally annotated.

## Methods

### Sample collection and ethics statement

An adult female specimens of *T. sibiricus* was originally collected from a forestry farm in Chifeng, Inner Mongolia Autonomous Region of China (41°39′N, 118°22′E) in October 2020. The sample was then maintained at Qufu Normal University, and stored at −80°C prior to DNA and RNA extraction. All experiments were performed according to the Guidelines for the Care and Use of Laboratory Animals in China. The sampled squirrel in this study was approved by the Institutional Animal Care and Use Committee (IACUC) of Qufu Normal University, Shandong, China, under permit No. 2021095.

### Sequencing

Muscle tissue of the female body was prepared for transcriptome, Illumina, PacBio whole-genome and Hi-C sequencing. All sequencing analyses were performed by the Shanghai Origingene Bio-pharm Technology Co. Ltd. (Shanghai, China). Genome DNA was extracted using a Blood & Cell Culture DNA Mini Kit (Qiagen, Germany). Quantity and quality of the total DNA were determined by 2100 Bioanalyzer (Agilent, USA) and Qubit 3.0 Fluorometer (Invitrogen, USA), respectively. Total RNA was isolated using a TRIzol Total RNA Isolation Kit (Takara, USA) following the manufacturer’s protocols^10^. The NanoDrop 2000 spectrophotometer (Labtech, USA) and 2100 Bioanalyzer were used to check RNA quality.

Whole-genome shotgun sequencing was performed with a single molecule real-time (SMRT) PacBio system. PacBio Sequel II libraries with an insert size of 30 kb were prepared using a SMRTbell Template Prep Kit 2.0. For survey analysis and the error rates associated with long reads, two short paired-end libraries with an insert size of 350 bp were constructed using Truseq DNA PCR-free Kit (Illumina, USA). The next-generation sequence data was generated on the Illumina Hiseq X10 platform. To construct pseudo-chromosomes, the Hi-C library was constructed according to the standard protocols described previously^11^. After quality control, 150 bp paired-end reads (PE150) were obtained using the Illumina Hiseq X10 platform. The cDNA library was constructed using a TruSeq RNA Sample Prep Kit v2 (Illumina, USA) and sequenced on the Illumina Hiseq X10 system using the paired-end strategy.

### Genome survey and assembly

**Fig. 1:**
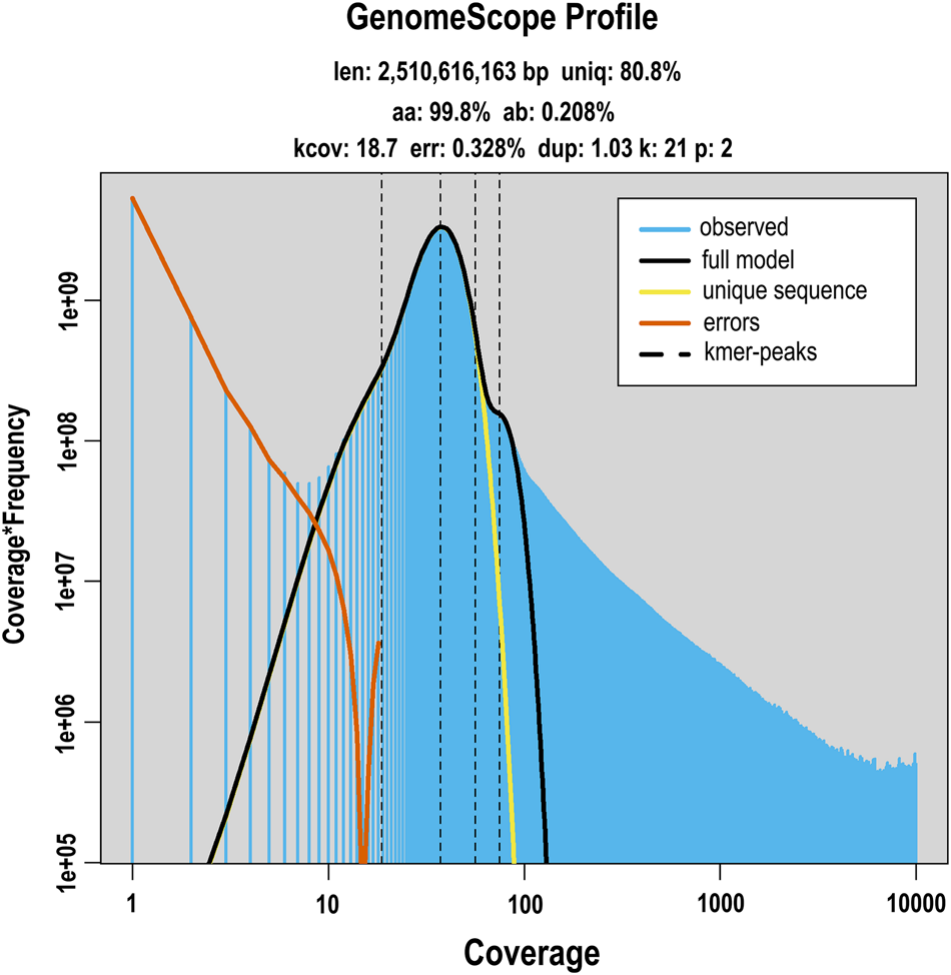
K-mer analysis of *Tamias sibiricus* genome.

A total of 132.39 Gb Illumina short-insert-size data was firstly generated to get a preliminary understanding of the genome characteristics (Table 1). Based on the clean data with duplications removed, the K-mer frequency distribution was calculated with Jellyfish v2.2.6^12^ and the results were subsequently analyzed by GenomeScope v2.0^13^. The genome size of *T. sibiricus* was estimated to be 2.51 Gb with the number of unique K-mers peaked at 21 (Fig. 1). Evaluation of genome characteristics showed the heterozygosity rate of the assembled genome was 0.21% (Table S1).

**Table 1:** Statistics of the DNA sequence data used for genome assembly.

Library	Insert size (bp)	Reads number	Raw data (Gb)	Average length (bp)	N50 length (bp)
Illumina	350	882,583,286	132.39	150	/
PacBio	30,000	4,625,634	111.63	24,134	35,623
Hi-C	350	1,449,171,836	217.38	150	/
RNA-seq	350	12,955,837	3.89	150	/

**Fig. 2:**
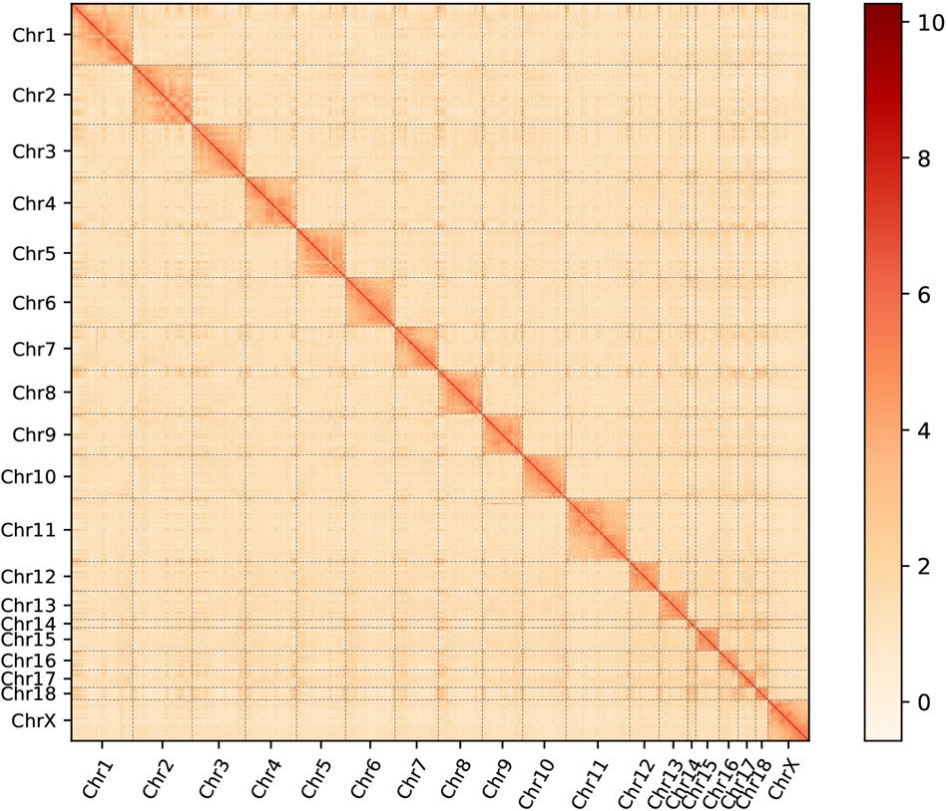
Heat map of Hi-C assembly of *Tamias sibiricus*. Color bar shows contact density from red (high) to white (low).

For PacBio sequencing, approximately 111.63 Gb long reads were obtained after removing adaptors in polymerase reads with default parameters. The mean length and N50 length of PacBio subreads was 35.62 and 24.13 kb, respectively (Table 1). After self-corrected and long read polished, genome initial assembly was performed using Canu v1.8^14^. As a result, we generated a 2.65 Gb genome assembly with the contig N50 of 9.40 Mb (Table 2). To further improve the quality and accuracy of the genome assembly, we corrected the genome by short-read polishing with high coverage of Illumina reads using Pilon v1.23^15^. Total size of the draft genome assembly was 2.64 Gb with an N50 length of 9.43 Mb. For the chromosome-level assembly, 217.38 Gb Hi-C sequencing data was generated and used to anchor contigs into pseudo-chromosomes (Table 1). 3D-DNA v180922 pipeline was used to generate a chromosome-level assembly of the genome^16^. After removing the duplicates, the Hi-C contact map was directly taken as input for 3D-DNA, the location and direction of each contig was determined, and the neighboring contigs were connected using 100 N gaps (100 Ns). Juicebox v1.11.08 (Juicebox Assembly Tools, JBAT) was subsequently used to review and manually curate scaffolding errors^17^. The final size of this genome was 2.64 Gb with a scaffold N50 of 172.61 Mb (Table 2). Results showed that the size of the assembled Siberian chipmunk genome was near to that estimated from the genome survey analysis. Meanwhile, 2.59 Gb data on the base level was anchored and orientated onto 19 chromosomes with a mounting rate of up to 98.03%, and the chromosome lengths ranged from 28.70 to 222.90 Mb (Table 3 and Fig. 2). After scaffolds were clustered, ordered and orientated to restore their relative locations, the heatmap of chromosome crosstalk indicated that the genome assembly was complete and robust (Fig. 1B).

**Table 2:** Summary of each step in construction of the *T. sibiricus* genome assembly.

Assembly	Total length (bp)	Number of scaffolds (chromosome)	N50 length (bp)	Longest scaffold (Mb)	GC (%)
Canu	2,654,018,856	5,517	9,396,557	66,610,541	40.2
Polish	2,643,565,565	4,100	9,431,264	66,852,084	40.4
3d-dna	2,643,804,365	2,097 (19)	172,614,981	222,896,756	40.4
Final assembly	2,643,804,365	2,097 (19)	172,614,981	222,896,756	40.4

**Table 3:** Statistics of chromosomal level assembly of *T. sibiricus*.

Chr ID	Chromosome length (bp)	Mapped Reads	Mean Depth
Chr1	215,335,440	613,008	34.818
Chr2	208,638,888	517,974	34.397
Chr3	187,191,955	1,483,941	36.714
Chr4	179,322,881	424,548	34.947
Chr5	172,614,981	532,782	35.870
Chr6	173,609,458	417,848	35.516
Chr7	152,265,710	404,153	35.788
Chr8	154,424,320	483,006	36.476
Chr9	141,821,005	373,157	36.299
Chr10	152,649,417	459,619	37.487
Chr11	222,896,756	621,036	35.761
Chr12	103,972,698	249,067	34.682
Chr13	100,012,300	769,173	35.762
Chr14	28,700,234	318,576	48.450
Chr15	82,000,469	203,418	35.026
Chr16	66,829,956	203,188	32.413
Chr17	61,561,036	136,250	32.504
Chr18	43,177,034	126,073	30.305
ChrX	144,589,955	449,466	38.154

### Chromosome synteny

**Fig. 3:**
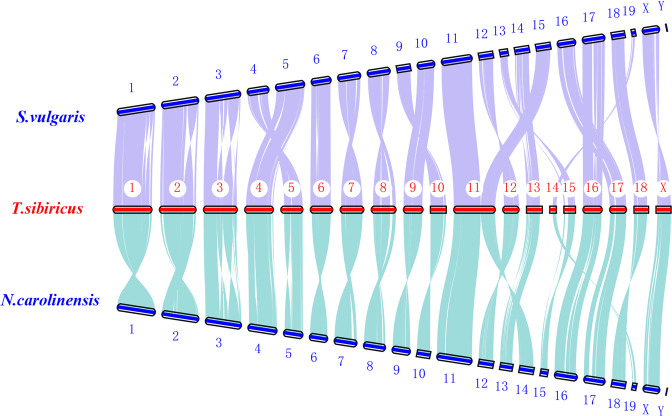
Genomic synteny between *Tamias sibiricus* and two other Xerinae species (*Sciurus vulgaris and Sciurus carolinensis*).

Collinearity analysis of chromosomes between *T. sibiricus* and two other Xerinae species (*Sciurus vulgaris* and *Sciurus carolinensis*) was conducted with LASTZ v1.02.00^18^. As shown in Fig. 3, all 19 pseudochromosomes of *T. sibiricus* displayed high homology with the corresponding chromosomes of another two squirrels, and two chromosomes (chr11 and chr15 of *S. vulgaris*, chr11 and chr14 of *S. carolinensis*) were fused to the chromosome (chr11) in the Siberian chipmunk. Previous studies, using cross-species chromosome painting, showed that the diploid number of chromosomes vary among the species in the superorder Glires (Rodentia and Lagomorpha)^19,20^, with the Siberian chipmunks having 38 chromosomes^9^. Interestingly, the variation seems to follow a certain pattern, such as chromosome 32,34,36,38,40. Combine that with our results of chromosome synteny, chromosome fusions and fissions might occur frequent among genome evolution of Glires. Thus, further studies are needed to determine the molecular mechanism of chromosomal rearrangements and evolution with more available chromosome-level genomic data.

### Repeat annotation

**Fig. 4:**
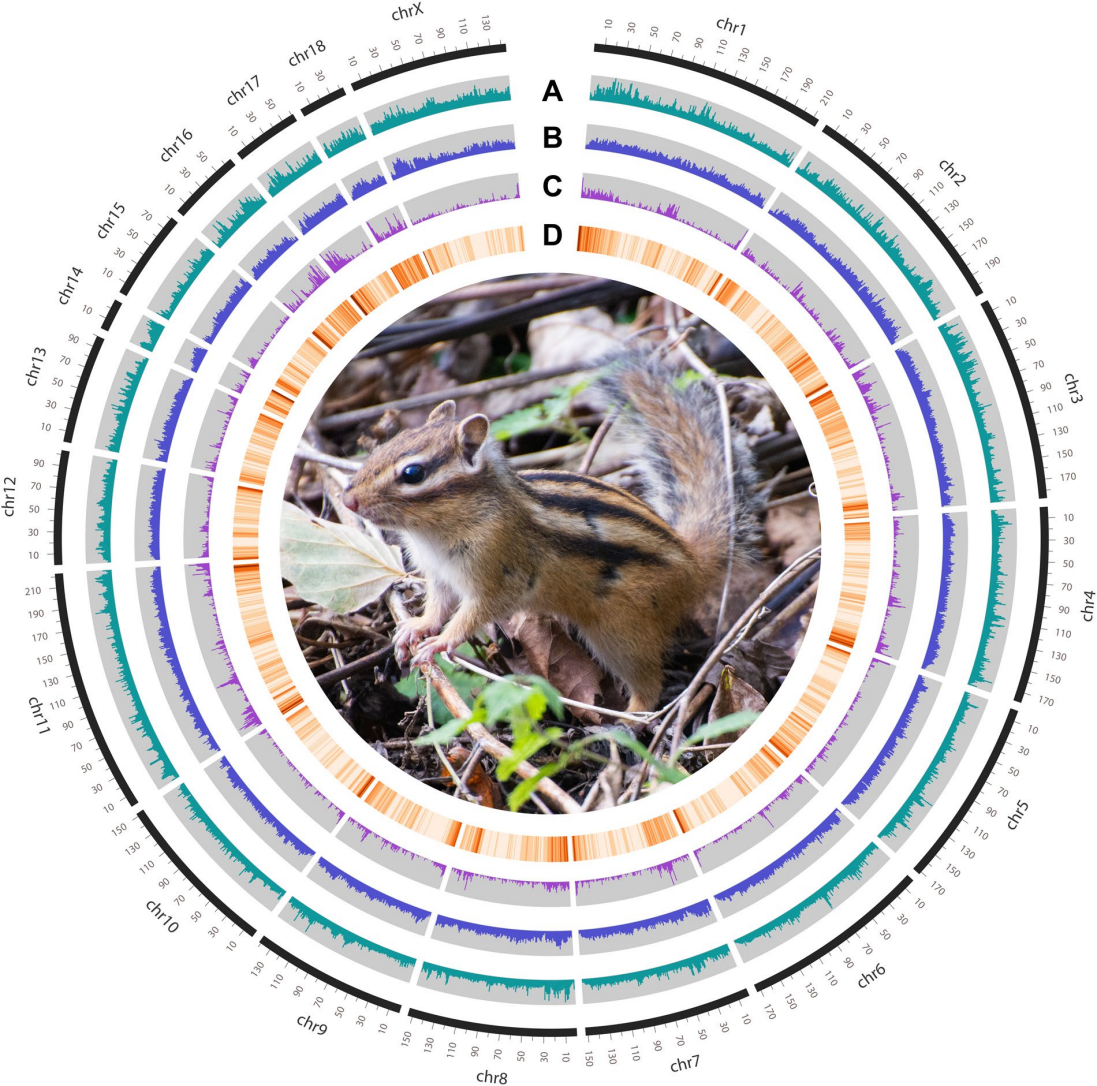
Genome characteristics of *Tamias sibiricus*. From the outer ring to the inner ring are the distributions of RNA TEs, DNA TEs, gene density and GC content.

After the genome assembly, annotation with 3 different types of repetitive sequences, non-coding RNAs (ncRNAs) and protein-coding genes (PCGs) was performed. RepeatModeler v2.0.1 was used to identify the repetitive elements with default parameters, and a *de novo* repeat sequence library was built using the results^21^. Then, a custom library was constructed combining with Dfam 3.1^22^ and RepBase 20181026 databases^23^. For the homology prediction, repetitive elements were masked using RepeatMasker v4.1.0 on the custom library^24^. A total of 1.03 Gb repetitive sequences were identified, constituting 38.87% of *T. sibiricus* genome. The predominant four categories of transposable elements (TEs) consisted of long interspersed nuclear elements (LINEs, 18.63%), DNA transposon elements (2.71%), long terminal repeats (LTRs, 10.11%), and short interspersed nuclear elements (SINEs, 8.90%) (Table 4 and Fig. 4). All ncRNAs (rRNAs, snRNAs and miRNAs) were annotated using Infernal v1.1.3^25^ and tRNAscan-SE v2.0.7^26^. Only high-confidence tRNAs were retained using the tRNAscan-SE script ‘EukHighConfidenceFilter’. Different types of noncoding RNAs (ncRNAs) were also annotated, yielding 6,265 tRNAs, 830 small nuclear RNAs (snRNAs), 92 ribosomal RNAs (rRNAs) and 595 micro RNAs (miRNAs) (Table S2).

**Table 4:** Repeat annotation in the *T. sibiricus* genome.

Type	Repbase TEs		*De novo*		Combined TEs	
	Length (bp)	% in genome	Length (bp)	% in genome	Length (bp)	% in genome
DNA	53,133,841	2.01	39,586,199	1.49	71,847,885	2.71
LINE	295,324,720	11.17	411,023,963	15.54	492,793,770	18.63
SINE	189,520,761	7.16	174,837,800	6.61	235,352,375	8.90
LTR	186,959,109	7.07	191,683,096	7.25	267,510,391	10.11
Unknown	2,074,690	0.07	37,565,203	1.42	39,637,495	1.49
Total	815,650,790	30.85	82,3843,171	31.16	1,027,834,241	38.87

### Protein-coding gene annotation

MAKER v3.01.03 pipeline was used to predict protein-coding genes with an integration of three strategies, including *ab initio* prediction, transcriptome-based annotation and homology-based annotation^27^. The *ab initio* prediction was generated using the pipeline BRAKER v2.1.5^28^, which automatically trained the predictors Augustus v3.3.4^29^ and GeneMark-ET^30^, and made use of the mapped transcriptome data and protein homology information. The transcriptome information in BAM alignments was produced by HISAT2 v2.2.0^31^, and the protein sequences were extracted from the database OrthoDB10 v1^32^. For transcriptome-based annotation, the data of RNA-seq was firstly mapped to our assembly with HISAT2, and the transcriptome information in BAM alignments was produced. With the reference genome of our assembly, the RNA-seq data were further assembled into transcripts using StringTie v2.1.4^33^. Protein sequences of five model rodentian species (*Cricetulus griseus*, *Dipodomys ordii*, *Ictidomys tridecemlineatus*, *Marmota marmota* and *Rattus norvegicus*) were downloaded from NCBI Refseq database. And all sequences were used as reference required by MAKER for the homology-based prediction. Overall, 25,311 protein-coding genes were predicted with an average gene length of 32,936 bp. The average exon number per gene was 7.52, with average exon length of 171.85 bp, and average intron length of 4850.84 bp. The final gene models predicted above were then annotated using the non-redundant (NR) protein database of NCBI, Swissprot, Pfam, the Kyoto Encyclopedia of Genes and Genomes (KEGG) and Gene Ontology (GO) databases. In total, 23,995 (94.73%) were successfully annotated for at least one homologous hit by searching against these five public databases. Based on BUSCO analysis, 94.4% of the BUSCO database (mammalia_odb10) genes were identified (complete single-copy genes: 92.2%, fragmented genes: 1.5%), further underlining the accuracy and completeness of gene prediction.

### Gene family

OrthoFinder v2.3.8 was used to inferred gene families (orthologue groups, orthogroups) with Diamond as the sequence aligner^34^. The protein sequences in the *T. sibiricus* genome and high-quality protein annotation sequences from assembled genomes of 19 rodents were used for analysis, including the naked mole‐rat (*Heterocephalus glaber*), Eurasian squirrel (*S. vulgaris*), eastern grey squirrel (*S. carolinensis*), alpine marmot (*M. marmota*), thirteen‐lined ground squirrel (*I. tridecemlineatus*), Arctic ground squirrel (*Urocitellus parryii*), Daurian ground squirrel (*Spermophilus dauricus*), Iberian mole (*Talpa occidentalis*), Ord’s kangaroo rat (*D. ordii*), European blind mole (*Nannospalax galili*), white-footed mice (*Peromyscus leucopus*), deer mouse (*Peromyscus maniculatus*), southern grasshopper mouse (*Onychomys torridus*), prairie vole (*Microtus ochrogaster*), Chinese hamster (*Cricetulus griseus*), golden hamster (*Mesocricetus auratus*), Norway rat (*R. norvegicus*), mouse (*Mus musculus*) and degu (*Octodon degus*). 20,952 gene families were identified among 20 species, and a total of 433,351 genes were obtained and assigned to the orthogroups (gene families) using OrthoFinder (Table S3). Gene family analysis also showed that the genes of single-copy orthologs was 5,277. Out of the 25,311 genes of *T. sibiricus*, 18,863 were clustered into 15,629 orthogroups, and 148 gene families and 502 genes were unique to *T. sibiricus*. The number of genes assigned to different orthologous groups was displayed in Fig. S1 and Table S4.

## Data Records

The genomic Illumina sequencing data was deposited in the NCBI Sequence Read Archive (SRA) database under accession No. SRR19929230^35^.

The genomic Pacbio sequencing data was deposited in SRA database under accession No. SRR19961223^36^.

The transcriptome Illumina sequencing data was deposited in SRA database under accession No. SRR19961278^37^.

The Hi-C sequencing data was deposited in SRA database under accession No. SRR19960530^38^.

The assembled genome was deposited in the GenBank at NCBI under accession No. GCA_025594165.1^39^.

Genome annotation information of repeated sequences, gene structure and functional prediction is available in the Figshare database^40^.

## Technical Validation

The completeness and accuracy of the assembled genome were evaluated using two different strategies. First, BUSCO analysis revealed that 92.9% (single-copied gene: 92.2%, duplicated gene: 0.7%) of 9226 single-copy orthologues (in the mammalia_odb10 database) were successfully identified as complete, 1.5% were fragmented and 5.6% were missing in the assembly (BUSCO v4.0.5). Second, we mapped the sequencing data to the assembled genome for verifying the accuracy. The mapping rates was 97.42%, 98.00% and 96.03% for the Illumina, RNA-seq and PacBio data, respectively.

## Supplementary information

Supplementary file 1

## Data Availability

No specific script was used in this work. The codes and pipelines used in data processing were all executed according to the manual and protocols of the corresponding bioinformatics software.

## References

[1] Wilson, D. E. & Reeder, D. M. *Mammal Species of The World. A Taxonomic and Geographic Reference*. (Smithsonian Institution Press, (1993).

[2] Oshida T, Masuda R, Yoshida MC (1996). Phylogenetic relationships among Japanese species of the family Sciuridae Mammalia, Rodentia), inferred from nucleotide sequences of mitochondrial 12S ribosomal RNA genes. Zool. Sci.

[3] Lee SJ (2011). Genetic origin identification of Siberian chipmunks (*Tamias sibiricus*) in pet shops of South Korea. Anim. Cells Syst..

[4] Chapuis JL (2005). Distribution in France of a naturalized pet, the Siberian chipmunk (*Tamias sibiricus*). Revue d’Ecologie.

[5] Wang Z-Y (2017). Scatter-hoarding behavior in Siberian chipmunks (*Tamias sibiricus*): An examination of four hypotheses. Acta Ecol. Sin.

[6] Kawamichi M (1996). Ecological factors affecting annual variation in commencement of hibernation in wild chipmunks *Tamias sibiricus*. J. Mammal..

[7] Marsot M (2013). Introduced Siberian chipmunks (*Tamias sibiricus barberi*) contribute more to Lyme borreliosis risk than native reservoir rodents. PloS One.

[8] Pisanu B, Obolenskaya EV, Baudry E, Lissovsky AA, Chapuis JL (2013). Narrow phylogeographic origin of five introduced populations of the Siberian chipmunk *Tamias* (*Eutamias*) *sibiricus* (Laxmann, 1769) (Rodentia: Sciuridae) established in France. Biol. Invasions.

[9] Beklemisheva VR (2011). Reconstruction of karyotype evoluti1on in core Glires. I. The genome homology revealed by comparative chromosome painting. Chromosome Res..

[10] Rio DC, Ares M, Hannon GJ, Nilsen TW (2010). Purification of RNA using TRIzol (TRI reagent). Cold Spring Harbor Protocols.

[11] Belton JM (2012). Hi-C: a comprehensive technique to capture the conformation of genomes. Methods.

[12] Marçais G, Kingsford C (2011). A fast, lock-free approach for efficient parallel counting of occurrences of k-mers. Bioinformatics.

[13] Vurture GW (2017). GenomeScope: fast reference-free genome profiling from short reads. Bioinformatics.

[14] Koren S (2017). Canu: scalable and accurate long-read assembly via adaptive k-mer weighting and repeat separation. Genome Res..

[15] Walker BJ (2014). Pilon: An integrated tool for comprehensive microbial variant detection and genome assembly improvement. PLoS One.

[16] Dudchenko O (2017). De novo assembly of the *Aedes aegypti* genome using Hi-C yields chromosome-length scaffolds. Science.

[17] Durand NC (2016). Juicer provides a one-click system for analyzing loop-resolution Hi-C experiments. Cell Syst..

[18] Harris, R. S. Improved Pairwise Alignment of Genomic DNA. Ph.D. dissertation, The Pennsylvania State University, Pennsylvania (2017).

[19] Li TL (2004). Evolution of genome organizations of squirrels (Sciuridae) revealed by cross-species chromosome painting. Chromosome Res..

[20] Li TL, Wang JZ, Su W, Nie WH, Yang F (2006). Karyotypic evolution of the family sciuridae: inferences from the genome organizations of ground squirrels. Cytogenet. Genome Res..

[21] Flynn JM (2020). RepeatModeler2 for automated genomic discovery of transposable element families. Proc. Natl. Acad. Sci. USA.

[22] Hubley R (2016). The Dfam database of repetitive DNA families. Nucleic Acids Res..

[23] Bao W, Kojima KK, Kohany O (2015). Repbase Update, a database of repetitive elements in eukaryotic genomes. Mobile DNA.

[24] Smit, A. F., Hubley, R. & Green, P. *Repeat Masker Open-4.0*. http://www.repeatmasker.org (2015).

[25] Nawrocki EP, Eddy SR (2013). Infernal 1.1: 100-fold faster RNA homology searches. Bioinformatics.

[26] Lowe TM, Eddy SR (1997). tRNAscan-SE: a program for improved detection of transfer RNA genes in genomic sequence. Nucleic Acids Res..

[27] Holt C, Yandell M (2011). MAKER2: an annotation pipeline and genome-database management tool for second-generation genome projects. BMC Bioinformatics.

[28] Hoff KJ, Lange S, Lomsadze A, Borodovsky M, Stanke M (2016). BRAKER1: unsupervised RNA-Seq-based genome annotation with GeneMark-ET and AUGUSTUS. Bioinformatics.

[29] Stanke M, Steinkamp R, Waack S, Morgenstern B (2004). AUGUSTUS: a web server for gene finding in eukaryotes. Nucleic Acids Res..

[30] Brůna T, Lomsadze A, Borodovsky M (2020). GeneMark-EP+: eukaryotic gene prediction with self-training in the space of genes and proteins. NAR Genomics Bioinf..

[31] Kim D, Paggi JM, Park C, Bennett C, Salzberg SL (2019). Graph-based genome alignment and genotyping with HISAT2 and HISAT-genotype. Nat. Biotechnol..

[32] Kriventseva EV (2019). OrthoDB v10: sampling the diversity of animal, plant, fungal, protist, bacterial and viral genomes for evolutionary and functional annotations of orthologs. Nucleic Acids Res..

[33] Kovaka S (2019). Transcriptome assembly from long-read RNA-seq alignments with StringTie2. Genome Biol..

[34] Emms DM, Kelly S (2019). OrthoFinder: phylogenetic orthology inference for comparative genomics. Genome Biol..

[35] (2022). NCBI Sequence Read Archive.

[36] (2022). NCBI Sequence Read Archive.

[37] (2022). NCBI Sequence Read Archive.

[38] (2022). NCBI Sequence Read Archive.

[39] Yi X-F (2022). GenBank.

[40] Li R, Yi X-F (2022). figshare.

