# Supplementary material for: Chromosome-level genome assembly of the Siberian chipmunk (Tamias sibiricus)
Source: Sci Data. 2022 Dec 24;9:783. doi: 10.1038/s41597-022-01910-5 (PMC9790022; doi:10.1038/s41597-022-01910-5)
Supplement: Supplementary file 1 [file 41597_2022_1910_MOESM1_ESM.docx]

**Supplementary materials：**

| **Property** | **Minimum** | **Maximum** |
| --- | --- | --- |
| Homozygous (aa) | 99.7732% | 99.8104% |
| Heterozygous (ab) | 0.189591% | 0.226846% |
| Genome haploid length | 2,507,055,934 bp | 2,510,616,163 bp |
| Genome repeat length | 482,088,006 bp | 482,772,611 bp |
| Genome unique length | 2,024,967,928 bp | 2,027,843,551 bp |
| Model Fit | 85.2358% | 99.4195% |
| Read Error Rate | 0.327545% | 0.327545% |

**Table S1.** The information of genome survey analysis.

**Table S2.** Annotations of non-coding RNAs in the *T. sibiricus* genome.

| **Type** | **Number** | **Total length** | **Average length** |
| --- | --- | --- | --- |
| rRNA | 92 | 28308 | 307.7 |
| snRNA | 830 | 91104 | 109.76 |
| tRNA | 6265 | 444767 | 70.99 |
| miRNA | 595 | 48648 | 81.76 |

**Table S3.** Statistics of gene families.

| **Elements** | **Value** |
| --- | --- |
| Number of species | 20 |
| Number of genes | 433,351 |
| Number of genes in orthogroups | 416,945 |
| Number of unassigned genes | 16,406 |
| Percentage of genes in orthogroups | 96.21% |
| Number of orthogroups | 20,952 |
| Number of species-specific orthogroups | 1211 |
| Number of genes in species-specific orthogroups | 4945 |
| Number of single-copy orthogroups | 5,277 |

**Table S4.** Statistics of ortholog genes.

| **Species** | **Singlecopy orthologs** | **Multi-copy orthologs** | **Unique orthologs** | **Other orthologs** | **Unclustered orthologs** |
| --- | --- | --- | --- | --- | --- |
| *Cricetulus griseus* | 8994 | 3304 | 265 | 9356 | 409 |
| *Dipodomys ordii* | 9150 | 2362 | 167 | 7729 | 579 |
| *Heterocephalus glaber* | 9033 | 2613 | 94 | 7836 | 500 |
| *Ictidomys tridecemlineatus* | 8986 | 2978 | 15 | 8567 | 309 |
| *Marmota marmota* | 8871 | 3171 | 78 | 8326 | 634 |
| *Mesocricetus auratus* | 8973 | 3216 | 177 | 9168 | 322 |
| *Microtus ochrogaster* | 9156 | 2624 | 31 | 8260 | 182 |
| *Mus musculus* | 9159 | 2827 | 758 | 9552 | 556 |
| *Nannospalax galili* | 9091 | 2743 | 85 | 8650 | 635 |
| *Octodon degus* | 8831 | 3277 | 156 | 8215 | 308 |
| *Onychomys torridus* | 9065 | 2916 | 38 | 8903 | 332 |
| *Peromyscus leucopus* | 8717 | 3846 | 38 | 9662 | 350 |
| *Peromyscus maniculatus* | 8862 | 3505 | 62 | 9520 | 426 |
| *Rattus norvegicus* | 8995 | 3397 | 175 | 9401 | 562 |
| *Sciurus carolinensis* | 8810 | 4301 | 1223 | 7494 | 1316 |
| *Sciurus vulgaris* | 8523 | 4219 | 208 | 8844 | 585 |
| *Spermophilus dauricus* | 8673 | 3769 | 271 | 7569 | 728 |
| *Urocitellus parryii* | 9197 | 2217 | 8 | 7808 | 210 |
| *Talpa occidentalis* | 8761 | 3485 | 574 | 8710 | 487 |
| *Tamias sibiricus* | 8794 | 3039 | 522 | 6508 | 6448 |

**
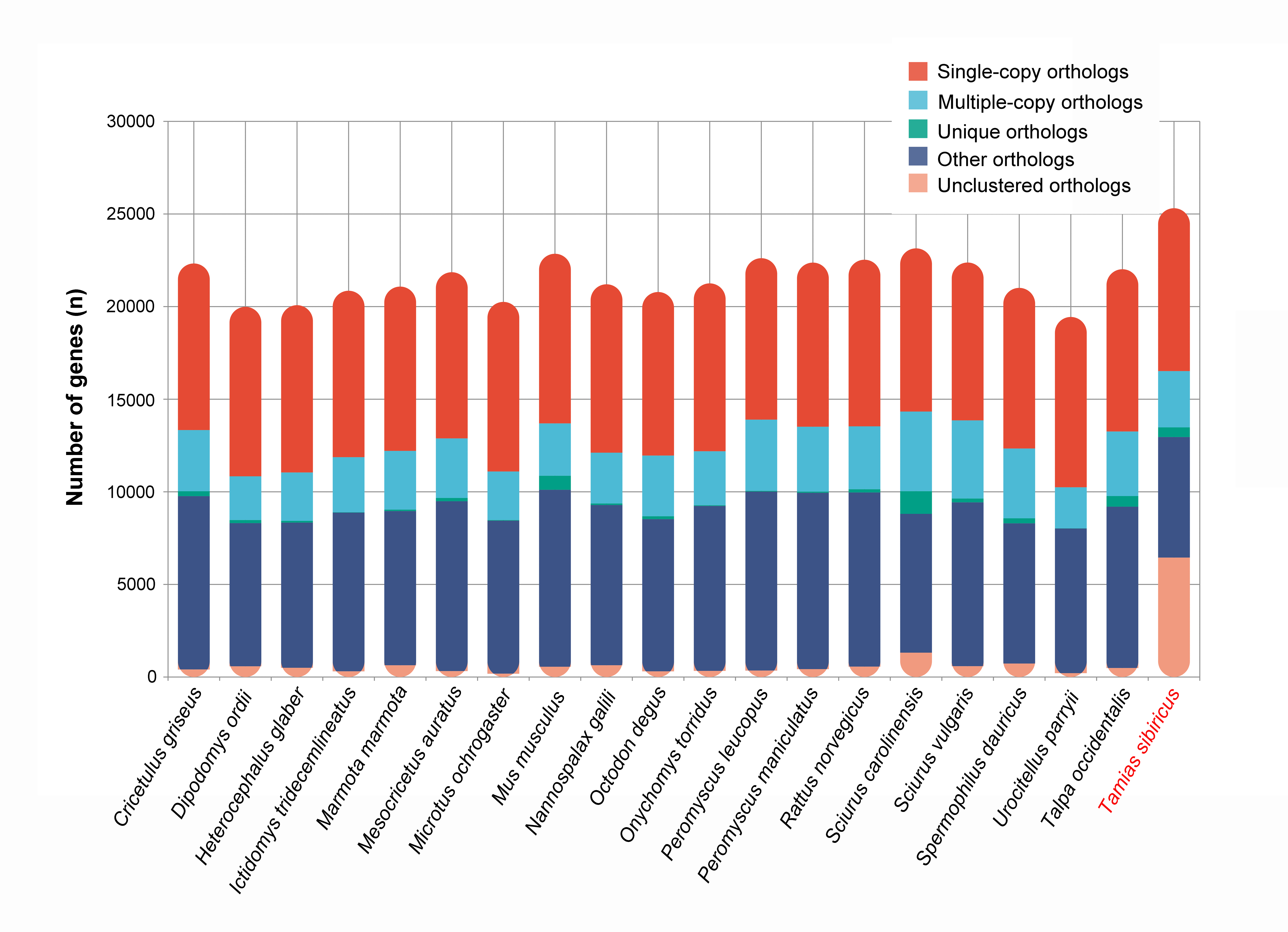
**

**Fig. S1** Comparison of copy numbers in gene clusters that reside in the genomes of *T. sibiricus* and 19 other Rodentia species. Single-copy orthologs denote that the family can have only one gene for each species, and multicopy orthologs denote that the family clustered more than one gene for each species. Other orthologs denote the family can have any number of genes for each species except the single-copy and multicopy orthologs. Unique paralogs denote species-specific gene families, and unclustered genes denote species-specific genes that cannot cluster with any other genes.
